# Excess mortality during the coronavirus pandemic (COVID-19) in Poland: Monika Burzyńska

**DOI:** 10.1093/eurpub/ckac131.197

**Published:** 2022-10-25

**Authors:** M Pikala, M Burzyńska

**Affiliations:** Department of Epidemiology and Biostatistics, Medical University of Lodz, Łódź, Poland

## Abstract

The COVID-19 pandemic, has begun a global changes in the mortality model, exceeding its predicted levels under standard conditions. The aim of the study was to assess the phenomenon of excess mortality in Poland in 2020 and in the first half of 2021 compared to 2016-2019 based on the data of the Central Statistical Office. The number of excess deaths was defined as the difference between the deaths in 2021 and the average number of deaths in the previous years. In accordance with the Eurostat methodology the 2016-2019 average was taken as the reference point. In 2020, the number of deaths in Poland amounted to 485,259 and was higher by 14.9% than expected on the basis of mortality in 2016-2019 (the absolute excess number of deaths amounted to 67,112). 43% of the excess deaths were deaths caused by Sars-Cov-2, 27% other deaths among infected people, and 30% deaths among those without confirmed infection. In this group, the highest increases were recorded for deaths due to cardiovascular diseases, neurological diseases and mental disorders. In the first half of 2021, 270,662 people died in Poland, i.e. 23.9% more than in the corresponding period in 2016-2019 and 22.9% more than in the first half of 2020, whereas 58,096 people died due to COVID-19 (22% of all deaths). The highest increases for non-viral deaths compared to 2016-2019 were recorded for blood diseases and immune mechanisms (121.53%), infectious and parasitic diseases (90.76%), mental disorders (34.93%) and cardiovascular diseases (11.65%). Excess mortality is a very serious problem of the public health. The increased mortality in 2020 and 2021 was closely related to the pandemic, as direct COVID-19 victims accounted for the majority of the observed increases in mortality. However, attention should be paid to the growth in mortality related to other causes, mainly mental disorders, for which mortality increases most rapidly, which requires immediate action.

**Key messages:**

• The increased mortality in 2020 and 2021 was closely related to the pandemic, as direct COVID-19 victims accounted for approximately 90% of the observed increases in mortality in Poland.

• In the group of no-related with COVID-19 deaths the highest increases were recorded for mortality due to cardiovascular diseases, neurological diseases and mental disorders.

